# Lyme Neuroborreliosis in a Patient with Breast Cancer: MRI and PET/CT Findings

**DOI:** 10.3390/diagnostics10010036

**Published:** 2020-01-09

**Authors:** Mathilde Ørbæk, Camilla Klausen, Anne-Mette Lebech, Helene Mens

**Affiliations:** 1 Department of Infectious Diseases, Rigshospitalet, Blegdamsvej 9, DK-2100 Copenhagen Ø, Denmarkhelene.mens@regionh.dk (H.M.); 2 Department of Radiology, Rigshospitalet, Blegdamsvej 9, DK-2100 Copenhagen Ø, Denmark; camilla.klausen@regionh.dk

**Keywords:** MRI, ^18^F-FDG PET/CT, lyme neuroborreliosis

## Abstract

We present a case demonstrating the performance of different radiographical and nuclear medicine imaging modalities in the diagnostic work-up of a patient with Lyme neuroborreliosis. The patient presented in late summer 2019 with radicular pains followed by a foot drop and peripheral facial palsy, both right-sided. Due to a history of breast cancer, disseminated malignant disease was initially suspected. Bone metastasis was ruled out by skeletal scintigraphy. Magnetic resonance imaging (MRI) of the neuroaxis and a whole body ^18^F-FDG PET-CT was performed within 48 hours. The MRI revealed a strong contrast enhancement of the conus medullaris and fibers of the cauda equina, while the ^18^F-FDG PET/CT was without pathological findings. Examination of cerebrospinal fluid led to the definitive diagnosis of Lyme neuroborreliosis with monocytic pleocytosis and a positive intrathecal test for *Borrelia burgdorferi*. The patient became pain-free after 10 days of ceftriaxone, and the paralysis slowly regressed the following month. This case highlights the difficulty of the diagnosis of Lyme neuroborreliosis and discusses the relevant imaging findings.

**Figure 1:**
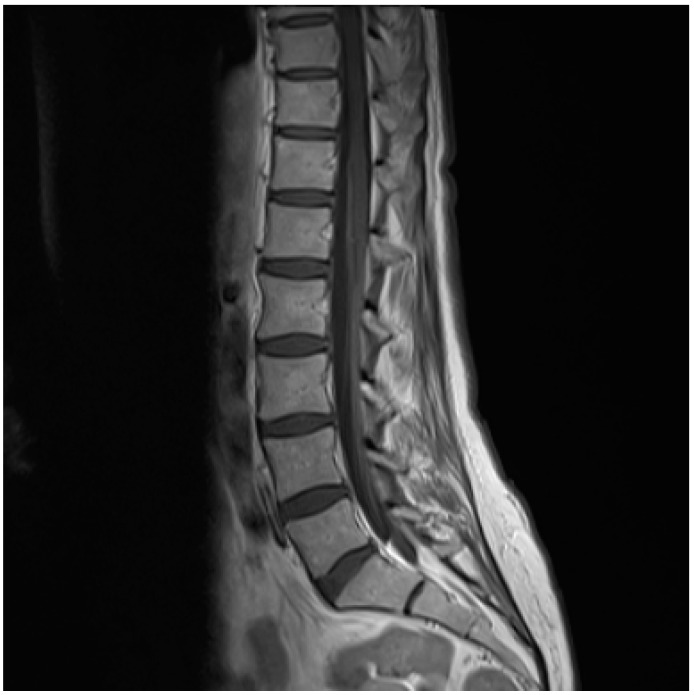
A 61-year-old female presented with opioid resistant radicular left-sided leg and back pains with nocturnal aggravation in July 2019. A year earlier she had been diagnosed with stage II breast cancer with lymph node metastasis. She had undergone lumpectomy with neoadjuvant chemotherapy, radiation and anti-estrogen treatment with letrozole. Initially, deep vein thrombosis was suspected; ultrasonic examination was performed, and anticoagulant therapy was initiated, as thrombosis could not be disproved. A skeletal scintigraphy conducted in the beginning of August was without evidence of bone metastases. Two weeks later the patient experienced progression of symptoms with band-shaped pain corresponding to Th8-10 and truncal dysesthesia, followed by unilateral foot drop and subsequent facial palsy, both right-sided. No cognitive deficiencies or nerve reflex abnormities were found. Under the suspicion of meningeal carcinomatosis, 150 mg prednisolone was initiated, and a magnetic resonance imaging (MRI) of the neuroaxis and a whole body ^18^F-FDG PET/CT was requested and performed within the next 48 hours. Laboratory testing was within normal range. White blood cells were 6.8 × 10^9^/L (normal range: 3.5–8.8 × 10^9^/L), hemoglobin 8.7 mmol/L (normal range 7.3–9.5 mmol/L), C-reactive protein 2 mg/L (normal range: 0–10 mg/L). Additionally, alanine aminotransferase (ALT), lactate dehydrogenase (LDH), bilirubin and creatinine were normal. The patient did not recall tick-bite or erythema migrans. However, due to signs of radiculopathy and mononeuritis, cerebrospinal fluid (CSF) was tapped and examined for inflammatory and malignant cells. The analysis showed CSF pleocytosis with 106 cells per µL (77% monocytes) and an elevated protein level of 1.14 g/L. CSF culture and polymerase chain reaction (PCR) for bacterial DNA in CSF were negative as well as PCR for herpes simplex virus, varicella zoster virus, enterovirus and cytomegalovirus. No malignant cells were detected in CSF, and flow cytometry was normal. The subsequent MRI showed a strong contrast enhancement of the conus medullaris.

**Figure 2:**
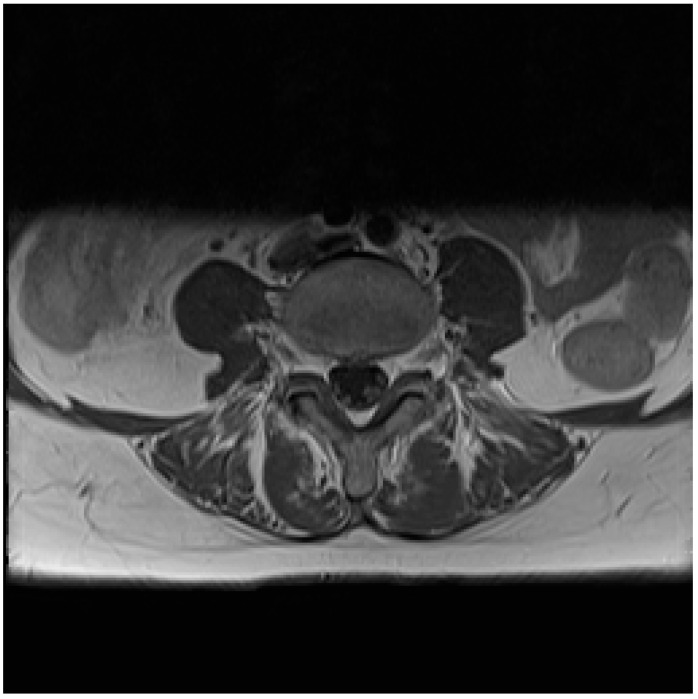
Furthermore, a strong contrast enhancement was shown on MRI of the fibers of the cauda equina and a discrete contrast enhancement of the cervical and thoracic nerve roots. The ^18^F-FDG PET/CT was without pathological findings. Intravenous ceftriaxone was initiated upon suspicion of Lyme neuroborreliosis and administered for a total of 14 days. *Borrelia burgdorferi (B. burgdorferi)* IgG antibodies were elevated in serum, and a positive intrathecal *B. burgdorferi* IgG antibodies synthesis was detected confirming the diagnosis of Lyme neuroborreliosis. The patient reported to be pain-free after 10 days of antibiotics, and the following month the paralyses almost completely subsided leaving only mild sensory disturbances in the left foot, as sequelae. Lyme borreliosis is a tick-borne infection caused by the *B. burgdorferi* sensu lato complex. *B. burgdorferi* can cause a variety of clinical manifestations including skin lesions, Lyme neuroborreliosis, Lyme carditis and Lyme arthritis [1]. The primary and most common manifestation is the skin lesion erythema migrans. Approximately 5–10% of individuals with an untreated erythema migrans (EM) develop Lyme neuroborreliosis, usually within 2–6 weeks. As illustrated within this case, only 25% and 50% recall a previous tick bite or erythema migrans, respectively, when they present with Lyme neuroborreliosis [2]. With an estimated prevalence of 3-120 per 100,000 inhabitants in Scandinavia, Lyme neuroborreliosis is one of the most prevalent bacterial infections of the nervous system [3,4]. The diagnosis of Lyme neuroborreliosis depends on classical symptoms including radicular pain and paralysis (typically facial paralysis), elevated white blood cells in CSF and the presence of intrathecal *B. burgdorferi* antibody production. *B. Burgdorferi* serum serology is not necessarily positive and should be interpreted with caution when suspecting Lyme neuroborreliosis [3]. Though adults with Lyme neuroborreliosis often manifest with a subacute painful meningoradiculitis and/or cranial nerve palsy, symptoms can appear in different stages and be clouded by comorbidities causing potential misdiagnoses and diagnostic delay. In these cases, imaging and especially MRI serve to assist with diagnosis [5]. A wide spectrum of imaging entities including diffuse affection in brain or spinal cord, meningeal and/or nerve enhancement and vascular affection has been demonstrated as well as overlap with findings consistent with multiple sclerosis [5,6,7,8]. Though none of these changes are pathognomonic for Lyme neuroborreliosis, they can serve as a contribution to diagnostics especially in case of nerval/meningeal enhancement, in which case lumbar puncture is recommended [5,9,10]. To our knowledge this is the first case presenting with definite Lyme neuroborreliosis where both MRI and ^18^F-FDG PET/CT was performed within 48 hours. Although MRI revealed an enhancement of the medulla as described above, no signal was detected by ^18^F-FDG PET/CT. Limiting data regarding findings on ^18^F-FDG PET/CT in patients with Lyme neuroborreliosis are available. PET/CT has revolutionized medical diagnosis in many fields by adding functional imaging to anatomic localization. The accumulation of ^18^F-FDG is dependent on the glycolytic activity of inflammatory cells but not an inflammation-specific tracer, as it accumulates in any cells using glucose as an energy source [11]. Two studies have investigated ^18^F-FDG PET/CT in patients with Lyme neuroborreliosis [12,13], one in patients with additional dementia [13]. Both studies primarily demonstrated cerebral hypometabolism, however others have casuistically described hypermetabolism and focal brainstem inflammation with corresponding neurologic symptoms [14,15]. Though useful in ruling out disseminated cancer disease, the ^18^F-FDG PET/CT did not reveal inflammation in nervous system, arguing for a limited use in the diagnostic work-up of Lyme neuroborreliosis. We speculate that the inflammation in Lyme neuroborreliosis might be driven by other cells than lymphocytes, for example macrophages, since it has as least once been described as dominating in single cases [16]. Different tracers targeting macrophages could be of interest in the visualization of *B. burgdorferi* infection in humans. In conclusion, this case illustrates the use and performance of different radiographic imaging modalities in the diagnostic work-up of a patient with a history of breast cancer diagnosed with confirmed Lyme neuroborreliosis, in which MRI and ^18^F-FDG PET/CT was performed within 48 hours. The diagnostic delay was primarily due to suspicion of disseminated malignant disease and the absence of tick bite in primary anamnesis. Bone scintigraphy and ^18^F-FDG PET/CT were both without pathologic findings. However, MRI revealed contrast enhancement of conus medullaris along with discrete cervical and thoracic enhancement, which to our knowledge is only reported in a few other cases with Lyme neuroborreliosis. This case and imaging findings serve as a strong indication of lumbar puncture (spinal tap) in order to rule out infection of the central nervous system.
